# Pennsylvania Hospital

**Published:** 1838-03-28

**Authors:** 


### PENNSYLVANIA HOSPITAL.

### TUMOURS.

*Wednesday, 21st March.* DR. RANDOLPH entered the amphitheatre at 11 A. M. and remarked.

We propose, to day, gentlemen, to perform an operation for the removal of a mammary gland, which is enormously enlarged. The patient is a female, forty-five years of age, who had about three years and a half ago, the first indications of a tumour on the left breast, which has since been gradually increasing in size, till it has become the immense mass, which will presently be shown you. It has not been a source of much pain, its chief inconvenience arising from its great bulk and weight. Nor has it caused any considerable derangement of the general health: you will indeed find the patient somewhat emaciated, but this is owing to the low diet to which she has been for a long time restricted—a rather improper plan of treatment, in my opinion. The external form of the tumour is irregular; it has the appearance of containing fluid, and is probably made up of several cysts. The greatest circumference of it measures twenty-one and a half inches. In making some remarks upon tumours a short time since, I stated to you, that a tumour properly speaking has been said to be a growth of a new production, and not a part of the original composition of the body. It may originate either from blood accidentally effused becoming organized, or from coagulated lymph deposited by a morbid action of the vessels. The size of the tumour becomes increased by depositions from the vessels which supply it with blood, but its structure and growth are modified also by the action which its own vessels assume, independently of the neighbouring vascular system.

Tumours sometimes become enormously enlarged. They differ very much from each other as to their structure. Thus, we have the fibrous, the adipose, the encephaloid or fungus hæmatodes, and the carcinomatous tumour. We have also, osseous tumours, such as, exostosis, spina ventosa, and osteo-sarcoma. Besides these, we have encysted tumours, as the atheromatous tumour, containing curdy matter; meliceritous tumour, containing matter resembling honey; and the steatomatous or fatty tumour. Encysted tumours vary as to their contents: these may be transparent, serous mixed with fibrin, sero-purulent, gelatinous, calcareous, &c. It rarely happens, that from the external aspect of the tumour, you can obtain a certain idea of its real nature. Sometimes however, from its feel and external character, you may form a correct diagnosis of its internal structure.

Tumours may be divided into solid and encysted. Most commonly solid tumours obtain a covering or envelope formed from the surrounding cellular membrane, which becomes condensed in proportion to the bulk of the tumour, and which completely isolates them from the healthy parts. In other instances however, tumours, especially those of a malignant character, have no such envelope, the tumour extending into and involving the neighbouring tissues.

Tumours bear often a very close resemblance to the structures in which they are found. Thus, we meet with adipose tumours situated in the cellular and adipose tissues; cartilaginous tumours may arise from the cartilages, and osseous tumours from the bones. At other times, however, there is no such analogy, and tumours are met with, differing not only in character from the tissues in which they are placed, but from every other known structure in the body.

A simple swelling, or enlargement of a part from deposition from the bloodvessels, arises from inflammatory action, resulting in the deposition of lymph. After the inflammation has subsided, the enlarged and debilitated vessels may not return to their original size, but continue to pour out fresh matter for the augmentation of the morbid mass. When however, the vessels are reduced, by natural or artificial means to their normal size, the tumour becomes arrested and may remain stationary, and the absorbents may gradually remove the new structure and restore the parts to their original condition. But, whenever deposition goes on in a ratio greater than that of absorption, the new structure becomes organized, and may attain a great size, and secrete from its own vessels a substance sui generis. After this it may change its character, lose all traces of the structure in which it originated, take on a new action peculiar to itself, and different from that of the adjacent part, and become converted into a tumour of the most injurious character. On a former occasion,* I made some observations on carcinomatous tumours, both in the indurated and ulcerated forms of the disease. You had before you last Wednesday, a patient from whose breast I removed a tumour: I trust that operation will prove instructive to you, for several reasons. I stated that I believed the tumour to be of a scirrhous character. I formed this opinion from its external appearance, as well as from the symptoms detailed by the patient herself, who stated, that she experienced at times severe lancinating pains through it. Upon cutting open the tumour after the operation, I ascertained that it did not possess a scirrhous character, but was an atheromatous tumour, consisting of a firm, curdy matter, enclosed in a dense cyst. From this result, we may deduce a strong argument for a preference of the knife to escharotics, in extirpating tumours of the breast. We thought the tumour in question to have been scirrhus. Suppose we had not cut into it. Our opinion would have remained the same, as well as the patient's, who would probably be aware of the extraordinary disposition of carcinomatous tumours to return. We should all have been under constant apprehension that this would be the case. The woman would have left this hospital, to all appearances, cured, but her friends would have said to her, yes, you have had your cancer cut out, but it returns nine times out of ten, and, in all probability such will be your case. If I had removed the tumour with caustic, inflammation and sloughing would have been the consequence, and the parts would have been so obscured, that the real nature of the disorder might have been concealed. We are now aware of the exact state of the case, and can give her strong assurance that she will go out cured, and remain permanently cured.

There is another view which may be taken of the matter. This patient will get well, as I said, permanently relieved. Now, had not the tumour been explored, after the operation, this case might be cited, many years hence, as a case of scirrhus completely cured by extirpation, whereas, it was not in fact a scirrhous tumour. I must here confess, that I entertain more favourable opinions of the efficacy of extirpation, for the relief of scirrhus, than many of my professional brethren. Some

* See No. 2, page 29.

surgeons consider the operation to be almost hopeless, so strong is the disposition of the disease to return. My own experience is rather contrary. I have witnessed and performed many operations, in which I have had no knowledge of the return of the disease. My friend, Dr. Brincklé, reminded me a day or two since, of an operation for the removal of a large scirrhous tumour from the mamma of a lady of this city, in the performance of which I aided him, ten years ago. The tumour was of great size, and Dr. Brincklé most happily and successfully removed it. The lady is still well, being up to the present time freed from a very aggravated form of the disease. What makes this case more striking, is, that her sister, who was similarly afflicted, died, refusing to submit to an operation. You may recollect the case of encephaloid tumour of the thigh,* in which I amputated the limb. The patient remains up to this time, quite well, and free from all appearances of a return of the disease, and she has been well a sufficient length of time, to prove the propriety of the operation. Upon that occasion I stated to you, that encephaloid or medullary sarcomatous tumours rarely were contained in a distinct cyst. They soon blend themselves with the adjacent parts, and the whole become involved in the same diseased action. Most commonly the medullo-sarcomatous tumour is subdivided by ligamentous bands, and the substance of the tumour consists of a matter resembling the brain, whence the tumour is termed encephaloid. In the commencement of the disease, the structure of the tumour is pretty dense, but after it has existed for some time, softening takes place to a greater or less extent, and in some instances would seem to indicate there was a large collection of purulent matter within the tumour. Pure pus is rarely found in such a tumour. Most commonly, in consequence of the process of softening, the bloodvessels give way, and we find masses of fibrine or fluid blood diffused throughout the substance of the tumour. After the tumour has existed for some time, the skin covering it becomes purple, and afterwards ulcerates. A fungus growth then protrudes, which sloughs readily and is speedily reproduced; in some instances impure pus is discharged. The bloodvessels which pervade the tumour, are most commonly obliterated by the effusion of coagulating lymph, and there is consequently but little hemorrhage. At other times considerble hemorrhage takes place, from the vessels giving way, and being left without the power to contract, from disease of their coats. At this period, the absorbents are contaminated, and the patient sinks rapidly, worn down by extreme exhaustion.

You saw, in the case alluded to, after the limb was removed, and the tumour exposed, that the whole of the tissues were involved, from the most exterior to the extreme interior, including skin, cellular substance, muscles, arteries, veins, ligaments, and bone. To such an extent was the thigh bone destroyed, that the patient fractured it in the simple act of walking up stairs.

The tumour that engages our attention to-day has been accurately described by Sir Astley Cooper; he terms it, the hydatid or encysted tumour of the breast. It differs from those I have enumerated in not possessing malignant properties; in not involving the absorbents, nor deranging the general health; in being confined to the mammary gland; remaining completely isolated, and manifesting no disposition to involve the surrounding tissue. If left to itself this tumour attains a great size. It is characterized by a distinct sense of fluctuation. This fluid contained is sometimes transparent, at other times slightly turbid. It is sometimes contained in a single cyst, but there are most commonly several cysts. Cases are recorded in which there was but a single cyst, cured by letting out the fluid, and introducing a tent. In this method inflammation is excited, and the cavity obliterated; the cure being effected after the manner of treating a hydrocele. Sir Astley Cooper mentions a case in which this method proved successful.

We will now have the patient brought in.

[Dr. Randolph next proceeded to perform the operation, present Drs. Harris, Barton, and Norris. Two semicircular incisions were made, and the tumour dissected out in fourteen minutes, the arteries being taken up as they were divided. Eight ligatures were put on. The usual dressings were applied. The tumour weighed, immediately after the operation, 4½ lbs. It was afterwards cut into, and found to contain several cysts; the mammary structure was much softened, and gave evidence that the process of degeneration had considerably advanced.]

* See No. 6, page 101.

